# Preface

**DOI:** 10.1007/s11154-022-09761-6

**Published:** 2022-11-02

**Authors:** Frederick CW Wu, Ilpo T Huhtaniemi

**Affiliations:** 1 Division of Endocrinology, Diabetes & Gastroenterology, School of Medical Sciences, Faculty of Biology, Medicine and Health, University of Manchester, M13 9WL Manchester, UK; 2 Professor Emeritus of Reproductive Endocrinology Institute of Reproductive and Developmental Biology, Department of Digestion, Metabolism and Reproduction, Hammersmith Campus Imperial College London, W12 0NN London, UK

This thematic issue on “Male Gonadal Function and Ageing” addresses an emerging and controversial topic of age-related changes in male gonadal function which may negatively impact the health and wellbeing of older men.

With the global increase in average life expectancy to 72 years or more, men are living almost a third of their lives after the age of 50. Increasing longevity however has not been accompanied by an extended period of good health. This has resulted in a burgeoning population of elderly men (and women) living for prolonged periods burdened with disabilities and chronic illnesses. Ageing healthily to break the relationship between chronological age and senescence through preservation of physical and mental quality of life (and avoiding or postponing the development of age-related comorbidities) in the elderly has become a major health target and societal concern [1].

Ageing in men is usually associated with a plethora of functional deteriorations and symptoms such as sexual dysfunction, prostatism, sleep disturbance, declining physical strength/function/mobility, falls/fractures, depression, cognitive impairment and accumulation of chronic diseases, culminating in frailty, dementia and loss of independence.

The expectation to defer or mitigate the above scenario of the decrepitude of ageing by preserving individual intrinsic capacity [2] and extending health span has revived interest in the pursuit of the ‘fountain of youth’ and the search for ‘elixirs of life’, where low-hanging fruits such as food supplements, fad diets, pharmaceuticals and hormones have increasingly attracted lay, medical, and scientific attention. The notorious exploits with animal testicular extracts in the 19th and early 20th centuries, initiated by Brown-Sequard, not only highlighted the overlap between the phenotype of senescence and clinical features of testosterone (T) deficiency, but also presaged the subsequent development of organotherapy and eventually synthetic T replacement. However, despite the undoubted health impact of oestrogen HRT for the female menopause, the more protracted decline in gonadal function in ageing men has received comparatively little attention until recently. Fuelled by the commercial incentive of an unexplored potential male HRT market, manufacturers of novel T preparations in the 1990s started to promote (by direct-to-patient advertisements or awareness campaigns to the medical profession) the concept of ‘Low T syndrome’ or the ‘male menopause’ in middle-aged and older men, who were drawn by the possibility of rejuvenation-like benefits akin to that experienced by menopausal women with oestrogen HRT and male hypogonadal patients from T replacement therapy.

Against this background, longitudinal population-based health surveys such as the Baltimore Longitudinal Study of ageing (BLAS) [3], Massachusetts Male Ageing Study (MMAS) [4], European Male Ageing Study (EMAS) [5] explored the possible relationships between hormone involution (the gonadal axis in particular) and a wide range of symptomatic, metabolic and functional deteriorations in ageing men. These paved the way for the first interventional randomised controlled trial (the T Trials) [6] to investigate the efficacy of T replacement therapy in symptomatic elderly men with low T.

Following up on some of the themes addressed in the landmark studies cited above, this special issue expands on the pathophysiology and clinical implications of the declining gonadal function in the ageing male. We have assembled an international panel of prestigious authors to critically appraise a wide-ranging collection of topics which addresses not only the latest conceptual and knowledge advances or gaps, but also controversies and debates. These state-of-the-art reviews will provide both trainees and experienced clinicians as well as academics with the best available evidence base with which to guide clinical practice and research in the management of the increasing population of ageing men with symptoms and functional deficits that could be related to low T concentrations. The following contains a brief introduction to the topics to be covered.

*Martins da Silva* and *Anderson* discuss the changes in endocrine and reproductive functions of men with ageing, focussing on changes in function of the hypothalamic-pituitary-testicular axis, affecting testicular T production and spermatogenesis. The decline in T levels occurs largely due to comorbidities, particularly obesity. The prevalence of sexual dysfunction increase with age while erectile dysfunction is a marker of arterial disease. While the male remains potentially fertile throughout his adult life, there are qualitative alterations in spermatogenesis with significant implications for offspring health.

The physiological mechanisms underlying the ageing-related decline of serum T concentrations, the health and lifestyle factors, the aging-related changes in T metabolism and androgen sensitivity associated with this decline are discussed by *Anawalt and Matsumoto*. Whether this ageing-related decline in serum T is a cause or a marker of poor health, and whether ageing-related male hypogonadism exists, are critically appraised to integrate available evidence into a rational clinical approach for the assessment and management of an older man with a low serum T concentration.

The hypogonadism of ageing men, in the absence of organic causes, is often referred to as functional or late onset hypogonadism (LOH). The review of *Kaufman* discusses the challenges of correct diagnosis of LOH. The man should present with a clinical syndrome suggestive of androgen deficiency and have consistently low serum T. However, multiple uncertainties are involved in LOH, such as the nonspecific nature of the signs and symptoms of androgen deficiency in older men, the clinical significance of moderately suppressed T levels, the role of associated comorbidities, and the debatable role of T therapy. A pragmatic and appropriately conservative approach is recommended in the diagnosis of LOH. The overall approach to diagnose the syndrome is in principle similar to the other forms of hypogonadism, but the danger of overdiagnosis should be emphasised above the risk of underdiagnosis.

T replacement therapy for older men with low T concentrations is a challenging but important issue faced by many clinicians and patients. Expanding on the data from the TTrials, *Snyder* discusses the benefits and potential risks of T treatment for LOH as well as clinical decision making arising therefrom.

The specific contribution of reduced T levels to the reduced sexual function of ageing men is still incompletely known. Likewise, the role of T replacement therapy, phosphodiesterase type 5 inhibitors, and their combination, in the treatment of erectile dysfunction are still controversial. *Corona* and *Maggi* summarize and critically appraise the role of low T and the currently available treatment options in the impairment of sexual function of ageing men.

Osteoporosis also affects men, and its prevalence is increasing with increased life expectancy, as discussed by *David, Narinx, Antonio, Evenepoel, Claessens, Decallonne* and *Vanderschueren*. The bone loss and increased fracture risk of older men is associated with the decrease of circulating androgen levels. Osteoporosis remains more often undiagnosed and untreated in men than in women. The authors provide several clinical and preclinical arguments in favour of a ‘bone threshold’ for the occurrence of hypogonadal osteoporosis. Although T replacement therapy increases the bone mineral density in men, no evidence for fracture risk reduction is available. T replacement therapy should not be used as the sole bone-specific treatment in ageing men.

One well-known aspect of ageing is the progressive decrease in skeletal muscle mass, strength, and power. The role of the ageing-related decline of T in sarcopenia and the role T supplementation in improving physical function and quality of life in older adults with functional limitations are discussed by *Gattu, Goldman, Guzelce, Galbiati* and *Bhasin*.

The review by *Welén* and *Damber* summarises the existing data on the associations between endogenous T and the development of benign prostate hypertrophy (BPH) and prostate cancer (PC). They conclude that there is no connection, and that androgen therapy could even improve PBH symptoms. Apparently, other factors than androgens are crucial for the development of PBH. In PC, circulating androgens are not either associated with the risk of initiation or progression of the disease. This apparent controversy could be related to the androgen receptor saturation model. In addition, the position of PSA measurements in the diagnosis and follow-up of men with T replacement therapy are discussed.

Low T is frequently associated with obesity/Metabolic Syndrome (MS)/Type 2 Diabetes Mellitus (T2DM). Potential mechanisms underlying the association between obesity and low T are complex. *Wittert* and *Grossmann* give a critical appraisal for two clinically-important questions, namely: the benefits and limitation of weight (including bariatric surgery) and lifestyle management for obesity/MS/T2DM in reversing low T or hypogonadism; the possible role for TRT in the management of Ob/MS/T2DM including effects on glucose metabolism (insulin sensitivity and glycaemic control) and body fat distribution.

Cognitive decline is an inevitable feature of ageing with major negative impact on quality of life. *Yeap* and *Flicker* discuss whether there is a clinically significant role of sex steroids in the occurrence, prevention, or treatment of cognitive decline and dementia in ageing men.

*Hauger, Saelzler, Pagadala and Paizzon* discuss the interaction of T levels with depression and the antidepressant effect of T treatment in hypogonadal men with depression. Major depressive disorders have clinically heterogeneous phenotypes with multifactorial and polygenic differences in pathophysiology. They reach the conclusion that the benefits of T replacement therapy on mood in men with clinically defined hypogonadism are uncertain.

The mass marketing of T products by direct-to-consumer product advertising (DTCPA) for common, non-specific, ageing-related symptoms associated with low T in older men has been regarded by some as disease mongering. Men’s health clinics have sprung up across the world and global T prescription have increased by some 20–fold into a multi-billion$ market in the last two decades. *Gagliano-Jucá & Basaria* give a critical and sobering appraisal of this phenomenon and provide helpful evidence-based advice for clinicians.

Males live shorter lives than women in all countries, and *Zhao* and *Crimmins* discuss the reasons for this universal phenomenon, which has not been succinctly characterized. The prevalence of lethal diseases is higher in men, shortening their life expectancy, whereas in women, non-lethal conditions prevail. Gender differences in the risk factors for disease have changed over time, and they are also influenced by gender-related differences in behaviour. Basic molecular and cellular measures related to ageing indicate that men age faster than women, but ageing is finally a combination of biology, behaviour, social and environmental factors.

As T is increasingly prescribed to older men around the world and it is also an integral part of developing hormonal male contraceptives for younger men, the potential risks of T treatment on cardiovascular disease are of critical clinical importance, but the topic remains highly controversial. *Thirumalai and Anawalt* synthesise the latest information (from epidemiological observational and interventional studies) and provides advice for clinicians regarding starting and continuing T therapy in patients with or without prevalent cardiovascular diseases.

Changes in sleep quantity and quality occur with ageing in men. *Liu and Reddy* discusses the possible relationships between sleep deficits and variations in T and other steroid hormones which may have important clinical implications, for example for shift workers. The relationships between obstructive sleep apnoea, obesity and T and the effects of T replacement therapy on obstructive sleep apnoea as well as the effects of continuous positive airways pressure (CPAP) treatment of obstructive sleep apnoea on T levels are also discussed.

*Carrageta, Guerra-Carvalho, Spadella, Yeste, Oliveira and Alves* review the animal models of male ageing and reproduction. Such studies are needed to complement human data, because relevant experimental approaches are needed to unveil the molecular mechanisms by which ageing affects the male reproductive potential. Such models are advantageous, including e.g. their reduced costs, general ease of maintenance in laboratory conditions, the availability of rigorous manipulation tools, the short lifespan of animals, their known genetic backgrounds, and reduced ethical constraints. The best known and widely applied reproductive ageing models are rodents and non-human primates. The data on testicular ageing, steroidogenesis, and genetic and epigenetic changes in spermatogenesis are detailed. Interestingly, some species challenge the current ageing theories and the concept of senescence itself.
